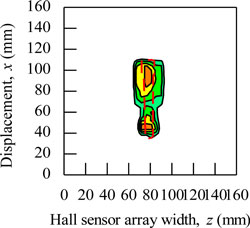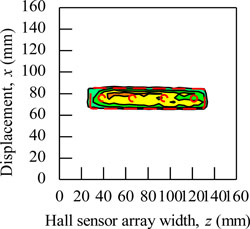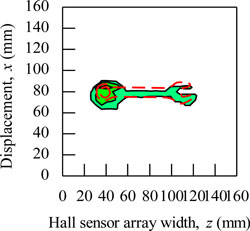# A Mobile Ferromagnetic Shape Detection Sensor Using a Hall Sensor Array and Magnetic Imaging

**DOI:** 10.3390/s111110474

**Published:** 2011-11-03

**Authors:** Norhisam Misron, Ng Wei Shin, Suhaidi Shafie, Mohd Hamiruce Marhaban, Nashiren Farzilah Mailah

**Affiliations:** Department of Electrical and Electronic Engineering, Faculty of Engineering, Universiti Putra Malaysia, Serdang 43400, Malaysia; E-Mails: weishin.ng@gmail.com (N.W.S.); suhaidi@eng.upm.edu.my (S.S.); hamiruce@eng.upm.edu.my (M.H.M.); nashiren@eng.upm.edu.my (N.F.M.)

**Keywords:** Mobile Hall Sensor Array, magnetic flux leakage, ferromagnetic material shape, magnetic imaging, magnetic flux distribution

## Abstract

This paper presents a Mobile Hall Sensor Array system for the shape detection of ferromagnetic materials that are embedded in walls or floors. The operation of the Mobile Hall Sensor Array system is based on the principle of magnetic flux leakage to describe the shape of the ferromagnetic material. Two permanent magnets are used to generate the magnetic flux flow. The distribution of magnetic flux is perturbed as the ferromagnetic material is brought near the permanent magnets and the changes in magnetic flux distribution are detected by the 1-D array of the Hall sensor array setup. The process for magnetic imaging of the magnetic flux distribution is done by a signal processing unit before it displays the real time images using a netbook. A signal processing application software is developed for the 1-D Hall sensor array signal acquisition and processing to construct a 2-D array matrix. The processed 1-D Hall sensor array signals are later used to construct the magnetic image of ferromagnetic material based on the voltage signal and the magnetic flux distribution. The experimental results illustrate how the shape of specimens such as square, round and triangle shapes is determined through magnetic images based on the voltage signal and magnetic flux distribution of the specimen. In addition, the magnetic images of actual ferromagnetic objects are also illustrated to prove the functionality of Mobile Hall Sensor Array system for actual shape detection. The results prove that the Mobile Hall Sensor Array system is able to perform magnetic imaging in identifying various ferromagnetic materials.

## 1. Introduction

This paper presents the development of a Mobile Hall Sensor Array (MHS) system for the detection of the shapes of ferromagnetic materials using a Non Destructive Evaluation (NDE) technique. NDE is a well-known technique used to describe and examine the condition of ferromagnetic materials without causing any damage to them. Furthermore, this NDE technique does not expose operator to health hazards. The NDE technique is often employed to evaluate the quality and condition of steel water pipes and steel cables embedded in walls or floors, as it is able to detect cracks and discontinuities within the material under evaluation. This NDE detection concept is based on the Magnetic Flux Leakage Test (MFLT) which requires the material under evaluation to be temporary magnetized either with permanent magnets or electromagnets. Thus, a magnetic flux flow is developed between the magnetic source (*i.e.*, permanent magnets or electromagnets) and the ferromagnetic materials under evaluation. The flux flow is detected quantitatively by magnetic sensors exposed to the magnetic flux. Any discontinuities or cracks observed in the ferromagnetic material will distort the magnetic flux flow detected by the magnetic sensors resulting in some magnetic flux leakage (MFL).

NDE techniques have also been widely used in pulse diagnostics, proximity sensing, and the evaluation of aircraft structures, train wheels, oil-gap pipes, power plants, chemical plants and nuclear plants. The purpose of these evaluations is to determine the quality of the surface under study; thereby allowing the necessary maintenance to prevent unwanted fatal accidents in advance. Today, many practical NDE ferromagnetic surface material evaluation techniques have been proposed in the industrial sector. In [1,2], a small yoke type electromagnet and a 181 differential type linearly integrated Hall sensor array design with a signal conditioning method was used for the evaluation of cracks in train wheels, while in [3], inductive sensor arrays placed on both sides of the train rails are used to detect the condition of train wheels as they pass. In [4], a flux set magnetic sensor is used for magnetic imaging of aircraft structure and laminated surfaces for critical defects. In another work [5–7], a magnetic camera with the magnetizer and 2-D Hall sensor array system has been proposed for magnetic imaging of ferromagnetic material surface cracks. Steel oil-gas pipeline’s surface condition has been evaluated in [8], using a yoke with permanent magnet and magnetic sensors as the sensor system and in [9], a 3D finite element method is used to calculate and model the magnetic flux leakage of a steel surface’s defects. In an oil well surface detection problem, a magnetic sensor system comprising a permanent magnet and a magnetic sensor along with the Gaussian Kernel RBF Neural Network is proposed for MFLT as discussed in [10]. For wire-rope surface defect detection, a circular 1-D Hall sensor array is employed to capture the 2-D magnetic leakage signals around the surface [11]. In [12], a Magnetic Anomaly Detection (MAD) Camera with a 2-D magnetic sensor array is developed to perform 2-D imaging to detect anomalies on the surface of ferrous objects. In oriental medicine applications, a system proposed in [13] comprising of a 2-D magneto-resistive sensor array with a permanent magnet is used for pulse diagnostic apparatus, whereby the pulses are translated from a 2-D magneto-resistive sensor array to the displacement of permanent magnet. In [14], a sensor system comprising a meander coil as sensor head and a pattern guide are used to detect displacement of moving parts in linear machines while in [15], a flexible Eddy current sensor array consisting of a forked coil array and a long flat cable is used for proximity sensing of gaps between curved surfaces. Last but not least, in [16], a magnetic rotation sensor consisting of a magnet, an aperture mechanism and a Hall sensor is used for accurate control of the amount of the transmitted light to a micro-display projection TV.

In this paper, a MHS system capable of visualizing the shape of ferrous objects through the magnetic imaging technique is presented. Various ferromagnetic materials of different shapes are placed beneath a non-ferromagnetic surface to mimic the actual application of detecting the presence of a ferromagnetic object placed in a walls or floor. The principle of operation for this MHS system is based on the MFLT, where the leakage flux from ferromagnetic object surfaces are detected and sampled. The sampled signals are then processed and the magnetic image for ferromagnetic object under evaluation is reproduced for validation. The MHS system is also capable of navigating along the x-axis through the ferromagnetic specimen and the evaluation is achieved with the development of magnetic images of the specimens under evaluation from the sampled 2-D array matrix of signals data.

This MHS system employs a linearly integrated 1-D Hall sensor array as signal sensing unit where the magnetic flux induced by a ferromagnetic specimen is detected and translated as Hall sensor output voltage signals. The 1-D Hall sensor output voltage signals from the Hall sensor array are processed with the developed data acquisition unit and data processing unit to construct the 2-D array matrix of Hall sensor output voltage signals for magnetic imaging. These 1-D Hall sensor output voltage signals captured by the data acquisition unit are then digitized by the A/D converter and transferred to the data processing unit set up in a netbook, which has the Hall signals array processing (H-SAP) application software to process the 1-D Hall sensor output voltage signals from the data acquisition unit. The H-SAP application software polls for data signals to be processed via the Universal Serial Bus (USB) communication terminal. In addition to signal processing, the H-SAP application software also employs an algorithm to construct the 2-D array matrix of Hall sensor output voltage signals for magnetic imaging. The magnetic imaging of ferrous object shapes is achieved using OriginPro 8^®^ software. Lastly, the experimental results presented in this paper show and prove the functionality of the proposed MHS system for ferrous object shape detection. The experiments have been carried out with both ferrous specimens and actual objects. The main purpose for ferrous object shapes detection on actual objects is to prove and verify the field applications of MHS system the detection of the dimensions of complexes shapes.

## 2. Structure and Basic Principle of the Mobile Hall Sensor Array

**Figure 1.**
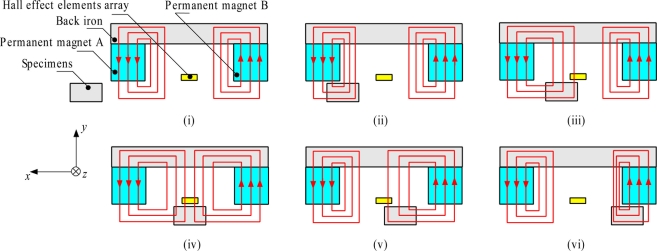
Structure and basic principle of the Hall Sensor Array.

The Hall Sensor Array consists of two permanent magnets A and B, back iron and an array of Hall sensors, as shown in Figure 1(i). The permanent magnet A is magnetized in a downward direction while the permanent magnet B is magnetized in an upward direction and the back iron is set at the top of both permanent magnet structures. A 1-D array of Hall sensors is placed in between these two permanents magnets. The concept of the MHS system detection is displayed in Figure 1.

Under normal conditions, the magnetic flux flow in the permanent magnets is as shown in Figure 1(i). As the ferromagnetic specimen moves along the *x*-axis, magnetic flux leakages (MFL) are developed and some of the magnetic flux flows through the specimen, as shown in Figure 1(ii). When the specimen reaches the 1-D Hall sensor array as illustrated in Figure 1(iii), the MFL is detected by the array. At this moment, a positive signal is produced by the Hall sensor due to the fact the MFL flows in an upward direction from the permanent magnet A. A higher MFL density value is detected as the specimen gets closer to the Hall sensor. When the specimen is at the center of the 1-D Hall sensor array as shown in Figure 1(iv), both MFL flows from permanent magnets A and B are detected by the 1-D Hall sensor array. However, due to the fact the MFL density flow is the same in opposite directions, the signals cancel each other out, so no signals are induced in the 1-D Hall sensor array. As the specimen continues to moves along the *x*-axis as in Figure 1(v), the MFL flow is in the downward direction through the permanent magnet B and is detected by the 1-D Hall sensor array. In this instance, a negative signal is generated by the Hall sensor. As the specimen moves away from the 1-D Hall sensor array as in Figure 1(vi), there will be no MFL flow detected, thus, no induced signals are developed by the sensor array.

**Figure 2.**
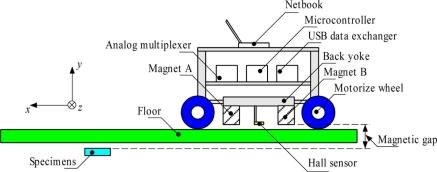
Structure of the Mobile Hall Sensor Array.

The structure of the MHS system is shown in Figure 2. Basically, this MHS system is a detection robot that includes a motor allowing it to move automatically during the inspection. The 1-D Hall sensor array is fitted on the lower level of the MHS system structure, as shown in Figure 2. In the second layer of the MHS system, the signal processing unit, including an analog multiplexer, microcontroller and USB data exchanger are located. The netbook is placed on the top layer for real time magnetic imaging monitoring. During inspection, the MHS system moves in the x-axis direction with a constant speed of 20 mm/s. The speed of movement is adjusted by controlling the speed of the motor attached to the wheel. The 1-D array of 33 Hall sensors is arranged in the z-axis direction with a total width of 140 mm. The specimen is placed under the floor/wall and the distance (magnetic gap in *y* direction) between the surface of the Hall sensor array and the surface of specimens is decided by the thicknesses of the floor/wall. In this study, a 10.0 mm magnetic gap is used. During the inspection, the MHS system navigates over the specimen and at the same time detects its shape.

## 3. System Operation, Signal Processing and Magnetic Imaging

### 3.1. System Operation

**Figure 3.**
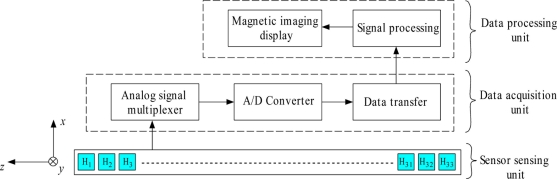
The basic operation of the Mobile Hall Sensor Array system.

Figure 3 shows the basic operation of the MHS system. The Hall sensor array is arranged along the *z*-axis of the sensor’s width direction, where each scanning inspection covers a width area of 140 mm. The sensor sensing unit comprises a Hall sensor array that converts the detected magnetic flux distribution to a voltage signal where the value is in accord with the range of magnetic flux magnitudes. The voltage signal of the Hall sensors, H_1_ to H_33_ are then sent to the data acquisition unit which consists of an analog signal multiplexer for data arrangement and is then digitized by the A/D converter.

When the conversion of Hall sensor voltage signals is complete, the digitized signals are arranged in a USB data packet and then sent to the data processing unit through the USB communication terminal. At the data processing unit, these signals are processed by the signal processing algorithm and appended to a 2-D array matrix. When the scanning is complete, the 2-D array matrix is entered to the OriginPro 8^®^ software to display a magnetic image of the scanned specimen.

**Figure 4.**
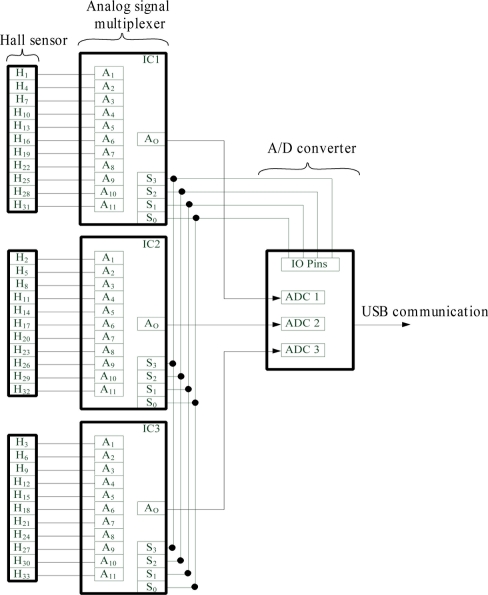
Connection of 1-D Hall sensor signals to analog multiplexer.

The analog signal multiplexer circuit channels the data from the signals of the 33 units of the 1-D Hall sensor array to the A/D converter embedded the microcontroller development board. Figure 4 below illustrates the connection of Hall sensor array signals from the 1-D Hall sensor array, H_1_ to H_33_ to the three analog multiplexers, IC1, IC2 and IC3, to be sent to A/D converter for the Analog-to-Digital conversion. The general purpose I/O pins on the microcontroller are configured to drive the three analog multiplexer for channels selection, namely, S_0_, S_1_, S_2_ and S_3_.

In the initial state of operation, the general purpose I/O pins from the microcontroller are used to drive the three analog multiplexer to select channel A_1_ or A_n_, where n is the channel number, from each multiplexer to output the three Hall sensor signals namely H_n_, H_n+1_ and H_n+2_ which in this case are H_1_, H_2_ and H_3_, to the A/D converter for digitization. The A/D converter in the microcontroller digitizes these signals, H_n_, H_n+1_ and H_n+2_ one by one from IC1 to IC3. As the A/D signal conversions are completed, the digitized signals, D_n_, D_n+1_ and D_n+2_ are then constructed in the USB data packet to be sent to computer through the USB communication terminal. As the data packet is sent, the microcontroller changes the output signals of the I/O pins to select the channel A_2_ of the three analog multiplexers. The process above continues until A_11_ of three analog multiplexers are selected. As the analog multiplexer reaches channel A_11_, the general purpose I/O pins of the microcontroller reset it back to A_1_. As the analog multiplexer channel selection resets back to A_1_, a new set of 1-D Hall sensor array signals is processed.

Table 1 shows the system specification configuration of the controller. A General Purpose Microprocessor (GPM) is employed for processing the signal array of the MHS system. The GPM is becoming popular in digital signal processing due to its high speed execution and availability at low cost [17,18]. A netbook with an Intel Atom N450 microprocessor operating at 1.66 GHz core frequency is employed for processing the signals of the 1-D Hall sensor array. The Signal Array Processing application software is developed to implement the signal processing algorithm defined for the Hall sensor array signal processing. Signal Array Processing is developed and compiled with Microsoft Visual Studio Professional 2005^®^. It is easily portable across multiple operating systems, including Windows XP, Windows 7 32-bit and Windows 7 64-bit. A Hall sensor with lower output voltage error tolerance e.g., Honeywell SS496A Solid State Hall Effect Sensor, is integrated into the 1-D Hall sensor array.

### 3.2. Processing of the Mobile Hall Sensor Array Signals

**Figure 5.**
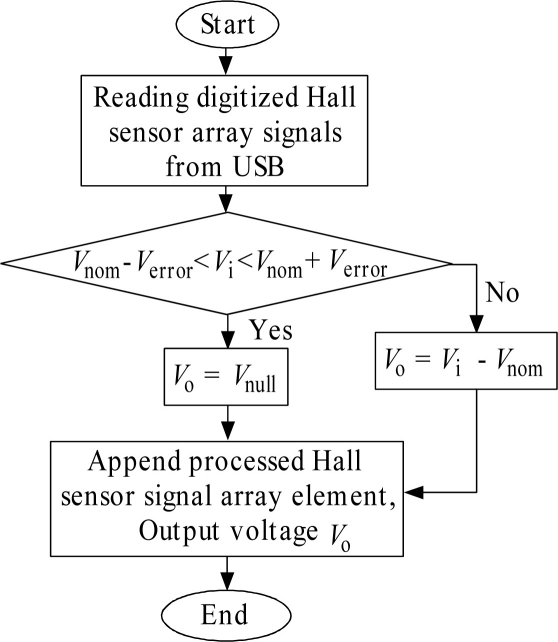
Flow chart of Hall sensor signal processing.

**Figure 6.**
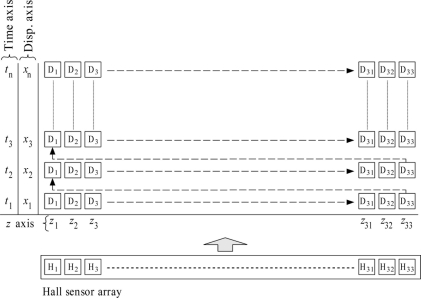
Data arrangement and construction in the Mobile Hall Sensor Array.

Figure 5 shows the Hall sensor signal processing flow chart. This flow chart explains the flow of the signal processing process on the data processing unit part, as shown in Figure 3. As explained in Figure 4, at any instant, three digitized Hall sensor signals, D_n_, D_n+1_ and D_n+2_ are transferred to the data processing unit through the USB communication terminal. As D_n_, D_n+1_ and D_n+2_ are transferred to the data processing unit, the algorithm shown in Figure 5 is carried out on D_n_ to D_n+2_ one by one. The three data signals are processed and the results are output as the 2-D array matrix, as shown in Figure 6. At this instant, the next three digitized Hall sensor signals data from the USB port are pulled to repeat the flow. In the data processing unit part, the general purpose microprocessor is used to process the signal since the microprocessor is becoming popular for digital signal processing due to its high speed execution core and low cost. This microprocessor is used for the data capturing, to process the signals that are produced by the 1-D Hall sensor array, and in the 2-D array signal matrix construction.

In the data processing unit, the digitized data from A/D converter received is produced by the Hall sensor array signals through the USB communication terminal between the microcontroller and the computer. The microcontroller is responsible for the A/D conversion of Hall sensor array signals. The A/D conversion is of 8-bit resolution. The calculation of voltage resolution for A/D conversion is shown in Equation (1):

(1) $$V_{\text{res}}=V_{\text{range}}/2N-1$$

where *V*_res_ is voltage resolution for A/D conversion, *V*_range_ is voltage range for A/D conversion and *N* is resolution bits for A/D conversion.

The quantization error in which is the difference between the original Hall sensor signals and the digitized signal is unavoidable with any voltage resolution in A/D conversions. However, the higher conversion resolution reduces the effect of quantization error. The maximum magnitude of quantization error for the Hall sensor array signals with A/D conversion is obtained based on Equation (3):

(2) $$V_{e}=V_{\text{res}}/2$$

where *V*_e_ is maximum magnitude of quantization error for A/D conversion.

In the A/D conversion of the Hall sensor array signals, the voltage resolution is of ∼0.02 V with the 0 V to 5 V voltage range for conversion. The maximum magnitude of quantization error derived from the voltage resolution is ∼0.01 V. The main purpose of this signal processing algorithm is to distinguish the Hall sensor array signal due to the presence of magnetized ferromagnetic shape from a normal condition. Then, the 2-D array is constructed from the real-time induced signals of the 1-D Hall sensor array. As mentioned earlier in this paper, the signal processing algorithm depends on the Hall sensor array signals under normal conditions. It uses these signals under normal conditions as a reference to distinguish the signals induced by the present magnetized ferromagnetic shape. Prior to performing the ferromagnetic shape evaluation, the Mobile Hall Sensor Array system is kept idle for 1 s for device initialization. During initialization, the reference signals for each Hall sensor are captured into the array. In the application, an array with 33 elements is defined to store the reference signal from each Hall sensor in the array. The Equations (3), (4) and (5) listed below are used for the implementation of the proposed signal processing algorithm:

(3) $$V_{\text{nom}}-V_{\text{error}}<V_{i}<V_{\text{nom}}+V_{\text{error}}$$

(4) $$V_{o}=V_{\text{null}}$$

(5) $$V_{o}=V_{i}-V_{\text{nom}}$$

where *V*_nom_ is the output voltage at normal condition, *V*_error_ is the output voltage error tolerance, *V*_i_ is the input voltage from the digitized Hall sensor signal, *V*_o_ is the output voltage offset from the nominal voltage and *V*_null_ is the null voltage (0 V).

Based on Equation (3) above, the output voltage error tolerance, *V*_error_ of each Hall sensor is the key parameter to determine the accuracy and sensitivity of the signal processing algorithm. It reads the output voltage of Hall sensors under normal conditions, *V*_nom_, as the reference voltage for signal processing. However, the signals within the output voltage error tolerance are not processed. The higher output voltage error tolerance results in a higher *V*_error_. Thus, this reduces the Signals Array Processing software’s ability to process the induced signals within the *V*_error_.

The flow chart in Figure 5 explains the signal processing algorithm for each Hall sensor linearly integrated into the array shown in Figure 6. In order to construct the processed signals of the Hall sensors array into a 1-D array, the algorithm shown in Figure 5 is repeatedly executed until the Hall sensor array signals from all the 33 channels are processed. This took approximately 200 ms to process and to construct the signal into 1-D array based on the time stamp routinely implemented within the Signals Array Processing application software. As the MHS system moves forward, a 2-D array is constructed from the 1-D processed signal array.

### 3.3. Data Construction and Magnetic Imaging Process

Figure 6 illustrates the top view of the 1-D Hall sensor array for ferromagnetic shape evaluation. The 1-D Hall sensor array signals are continuously appended to the new row of 2-D array at approximately 200 ms intervals. The width, *x*, of the array is fixed by the length of the 1-D Hall sensor array shown in Figure 3. However, the height, *y*, of the array increases as the Hall sensor array moves forward in the direction shown in Figure 6. The constructed 2-D array signals are output to a text file in matrix form which is used to construct the magnetic image of ferromagnetic shape under evaluation. In Figure 6, at an instant, the signal processing application on data processing unit gets completed with the processing of data signals, D_n_, D_n+1_ and D_n+2_. The processed signals are appended to the 2-D array matrix shown in Figure 6. The next data signals from USB communication continue to be processed and appended to the same row until it reaches D_31_, D_32_ and D_33_. As it reaches the D_31_, D_32_ and D_33_, the signals processing application append to the new row starting with D_1_, D_2_ and D_3_. The 2-D array matrix continues to get appended with new rows of processed data signals from D_1_ to D_33_ until the evaluation of the ferromagnetic shape is completed. As the ferromagnetic shape evaluation is completed, the 2-D array matrix shown in Figure 6 is fetched to the OriginPro 8^®^ application for magnetic imaging.

## 4. Magnetic Imaging Results and Discussions

**Figure 7.**
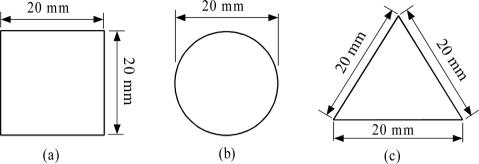
The shape and size of specimens, (**a**) Square shape; (**b**) Round shape; (**c**) Triangular shape.

Figure 7 shows the shape and size of the specimens used for magnetic imaging inspection verification. A square shape, round shape and triangular shape were used. The thickness of each of the specimens is 3 mm. The material for these specimens is a soft iron ferromagnetic (SS400).

The design setup to evaluate the shape of specimens with the MHS system is shown in Figure 2. The MHS system is moving at a constant speed of 20 mm/s during inspection of the ferromagnetic shapes. The specimens are placed underneath the non-ferromagnetic floor/wall separately in order to distinguish the magnetic image of various shape surfaces. The magnetic gap between sensor head and ferromagnetic surface is 10 mm. During the inspection, the MHS system is moved forward and the real-time signal array processing of the MHS is accomplished by connecting the mobile system to the netbook.

While the MHS is moving forward, the 1-D array signals are being continuously captured and appended into the 2-D array as explained in Figure 5. As the shape evaluation is completed, the 2-D array signals are output to a text file in a matrix form. The magnetic images of the ferromagnetic shapes are then plotted from the 2-D array signals matrix in this text file.

### 4.1. Data Distribution and Construction during Inspection

**Figure 8.**
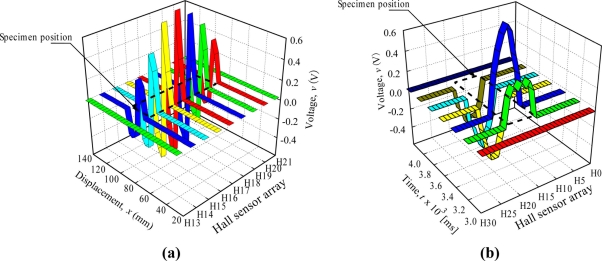
Data arrangement and distribution constructed with the H-SAP application based on 1-D Hall sensor array signals for a square shape specimen. (**a**) Voltage value arrangement and distribution based on the *z* axis of Hall sensor array direction and *x* axis of displacement. (**b**) Voltage value arrangement and distribution base on the time axis *t* and *z* axis of Hall sensor array direction.

Figure 8 shows the data arrangement and distribution constructed with H-SAP application based on 1-D Hall sensor array signals for the square shape specimen. Figure 8(a,b) are of the same data, but they are plotted from a different point of view. The dotted line shows the actual position of the square shape specimen. The dotted lines do not show a square shape because of graph scaling. In Figure 8(a), the voltage value is plotted based on the *z* axis of Hall sensor array direction and *x* axis of displacement. The voltage values for Hall sensor numbers H_13_ and H_21_ show no change because no detection of flux leakages is observed as the specimen did not pass through both of these elements, but for the Hall sensor numbers H_14_ to H_20_ the voltage values show changes in the displacement value of 62 mm to 82 mm. The voltage value starts to change from positive voltage at a displacement of 62 mm and become zero at 72 mm before going to a negative value to 82 mm, then back to a zero value. This phenomenon occurred as discussed in Figure 1, where the Hall Effect element detects the positive and the negative direction of flux leakage as the specimen passed through it. The voltage value is proportional to the magnitude of flux leakage, where the voltage value for Hall sensor numbers H_16_ and H_17_ show the highest value because the flux leakage is concentrated at the middle of the specimen. The Hall sensor numbers H_14_ and H_20_ exhibit small flux leakage values as they detect only the edges of the specimen.

In Figure 8(b), the voltage value is plotted based on the time axis *t* and *z* axis of Hall sensors array direction. This explains the data arrangement by the computer in the signal processing unit. The process of data collection from all 33 units of the Hall sensor array takes 200 ms to finish. For the first data array at *t* = 3 ms, no flux leakage is detected. The detection starts at *t* = 3.2 × 10^3^ ms with Hall sensor number H_14_ to H_20_ and achieves a maximum induced value at t = 3.4 × 10^3^ ms where the sensor array is at the middle of specimen. At *t* = 3.6 × 10^3^ ms the voltage value changes to negative until *t* = 4.0 × 10^3^ ms as the negative direction of flux leakage is detected.

**Figure 9.**
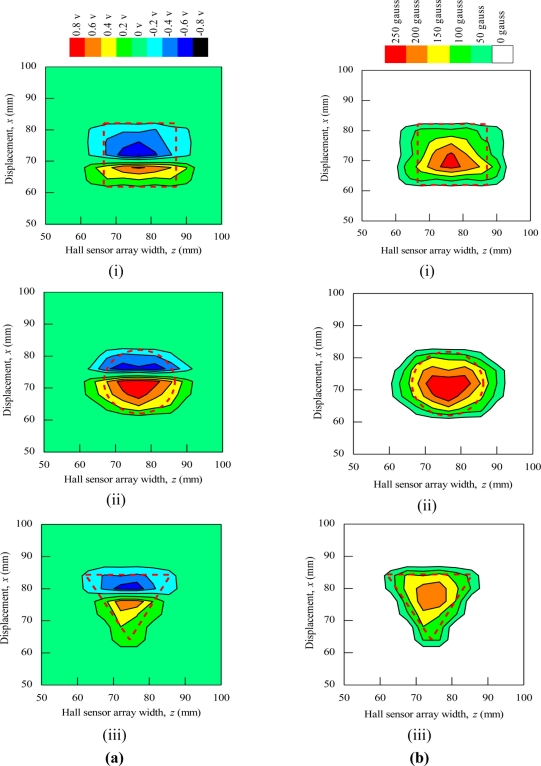
Magnetic imaging for square, round and triangular specimen shapes. (**a**) Induced Hall Voltage value distribution; (**b**) Absolute value of the flux density distribution.

During the magnetic imaging process, all the signals data from 1-D Hall sensor array acquired by the computer are arranged and plotted in 2-D image matrix form, as shown in Figure 9. The magnetic images in Figure 9(a) illustrate the voltage value with polarity due to detection of different direction of flux leakage from various ferromagnetic shapes as presented before. The dotted line shows the actual shape of the specimen. The shape of specimens is seen on the voltage distribution as: (i) square shape, (ii) round shape and (iii) triangular shape. The detection in the displacement direction *x* shows good imaging accuracy for the square shape and round shape where the MHS system properly detected the 20 mm length of both shapes, but the detection in the displacement direction *x* for the triangular shape imaging is of over 5 mm length.

Meanwhile, for the detection in the Hall sensor array width direction *z*, all shapes shows inaccurate image detection with 10 mm over the width of detection. This is because of the effect of flux leakages at shape edges and the size of each Hall sensor. A small sized Hall sensor is required to produce accurate image detection. Furthermore, in order to improve the imaging technique for ferromagnetic shapes, the magnetic image of various ferromagnetic shapes is proposed to be plotted by only considering the magnitude of induced voltage signals. From the magnitude of the induced voltage, the magnitude of the induced magnetic field can be obtained with Equation (6):

(6) $$\left|B_{\mathrm{ind}}\right|=1/3.125\left|V_{\mathrm{ind}}\right|$$

where *B*_ind_ is the magnitude of induced magnetic field in Gauss and *V*_ind_ is the magnitude of induced voltage in mV.

The magnetic image in Figure 9(b) illustrates the absolute value of flux density distribution in Gauss with a complete image of various ferromagnetic shapes. The dotted line shows the actual shape of each specimen. Complete and smooth magnetic images (no-gap in between magnetization polarity change) are constructed for various ferromagnetic shapes. The shapes can be clearly identified from the magnetic images. The magnitude of the magnetic field increases towards the center of ferromagnetic shapes. This implies that the magnetic flux distribution is more concentrated at the center of the ferromagnetic shapes. The overall results presented prove the ability of the MHS system for magnetic imaging of ferromagnetic shapes even when the Hall sensor array width *z* direction shows inaccurate detection.

### 4.2. Magnetic Imaging of Specimens

The functionality of the MHS system for shape detection is shown in Figure 9(b) with the soft iron (SS400) specimen described in Figure 6. In addition, the magnetic imaging on actual ferromagnetic objects with complex dimensions was also performed. The purpose was to assess the ability of the MHS system in the shape detection of ferromagnetic objects with complex dimensions for field applications. Table 2 presented some examples of the magnetic images for a pair of pliers, a key, a steel bar, a screwdriver, a steel plate and a wrench with absolute quantities. The dimensions for the pliers and screwdriver are bore shape, in which the thicknesses are inconsistent and in certain parts the thickness can reach up to 5 mm. On the other hand, shapes like the key, steel bar, steel plate and wrench are consistent in thickness but of different thickness from each other. The thickness of the wrench is 3 mm, while the thickness for the key, steel bar and steel plate is around 2 mm. Small holes exist in each ferromagnetic object.

Basically, the magnetic images shown in Table 2 are able to visualize the shapes of all actual ferromagnetic objects. However, for the pliers and screwdriver the magnetic images are distorted when compared to the original shapes. The magnetic images deviate from the actual dimensions due to the variable thicknesses of each object. This is because the variable thickness will make the flux leakage distribute inconsistently and the resulting magnetic imaging will deviate from the actual dimensions. On the other hand, for the magnetic images of a key, steel bar, steel plate and wrench shows almost the same shape as the originals with the actual external dimensions since their thickness is consistent. Unfortunately, the magnetic images for the small holes in the objects cannot be detected. This is because the size of the holes does not have a significant enough effect on the flux leakage along the *y* axis to be detected by the Hall sensors. To overcome the problem, the size of the Hall sensors and degree of surface slope are the main factors for detecting the *y* axis flux leakage in order to archive accurate magnetic images.

## 5. Conclusions

The development of a Mobile Hall Sensor Array for ferromagnetic material shape detection using the non-destructive evaluation concept was presented in this paper. This Mobile Hall Sensor Array system consists of a motorized structure, sensor and signal processing unit. The sensor has a simple structure with two permanent magnets A and B, back iron and an array of Hall sensors. The permanent magnet A is magnetized in the downward direction and the permanent magnet B is magnetized in the upward direction to produce the magnetic flux flow. The array of Hall sensors is used to detect the leakage flux that is present due to the shape of the ferromagnetic structures.

The signal processing unit processes the data as the Mobile Hall Sensor array system moves forward. The 2-D array matrix for magnetic imaging is then constructed from the 1-D processed signals array captured by the Hall sensors. From the 2-D array matrix, the detection of the ferrous objects’ shape is determined using the algorithm developed for this application.

Experiments using ferromagnetic material specimens of different shape and actual ferromagnetic objects have been done to evaluate the functionality and performance of the Mobile Hall Sensor Array system. For ferromagnetic shape specimens, the results for shape detection in the *x* displacement direction show good accuracy with the magnetic images of said specimen. But, for the detection of width in the *z* direction, all shapes shows inaccurate image detection with 10 mm over width of detection. This is because of the effect of flux leakages at shape edges and the size of each Hall sensor. A small Hall sensor size is required to produce accurate image detection. The magnetic imaging on actual ferromagnetic objects with complex dimensions was also performed. The magnetic images obtained show that the sensors are able to visualize the shapes of actual ferromagnetic objects, but the magnetic imagining of the objects deviated from their actual dimensions due to the variable thickness of each object. This is because the variable thickness will make the flux leakage distribute inconsistently, thus resulting in the magnetic imaging deviating from the actual dimensions. From the overall presented experimental results, the MHS system is still able to perform magnetic imaging on various ferrous object shapes.

## Figures and Tables

**Table 1.** System Configuration Specifications.

**Item part**	**Specification**
Hall sensor	Honeywell SS496A Solid State Hall Effect Sensor Dimension: 4 mm (w) × 3 mm (h) Supply Voltage: regulated 5 V Output Operating Range: 0.5–4.5 V
Analog signal multiplexer	Burr Brown MPC506A 16-Single Ended Analog Multiplexer Reference Voltage: internal regulated 5 V Output Operating Range: 0–5 V Switching Time: 5 μs Supply Voltage: regulated +/−10 V
A/D converter and USB Communication	ATMEL AVR AT90USB646 Memory: 64 K In-system Self Programmable Flash I/O: 48 Programmable IO lines A/D: Up to 8 A/D converter channel with 10 bits resolution A/D Conversion Time: 200 μs USB Device: Low Speed and Full Speed data transfer Supply Voltage: 5 V
Signal processing and Magnetic Imaging	Dell Mini 10 Netbook Processor: Intel Atom N450 1.66 GHz with 512 K L2 Cache Memory: 2 GB DDR2 SDRAM USB: 4 USB2.0 ports supporting Low, Full and High Speed Operating System: Windows 7 Basic

**Table 2.** Comparison of actual images and magnetic imaging of actual ferrous objects.

	Plier	Key	Steel bar
Actual image	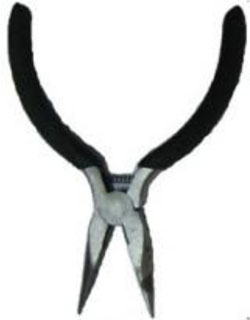	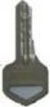	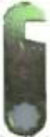
Magnetic imaging	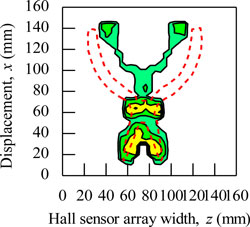	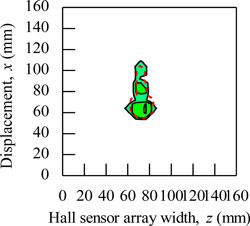	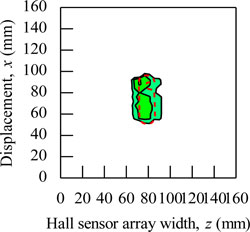
	Screw driver	Steel plate	Wrench
Actual image	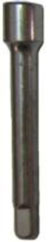	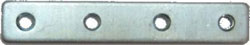	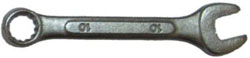
Magnetic imaging